# Food choice patterns of long-haul truck drivers driving through Germany, a cross sectional study

**DOI:** 10.1186/s40795-019-0326-3

**Published:** 2019-11-26

**Authors:** Andreas Bschaden, Siegfried Rothe, Anja Schöner, Nina Pijahn, Nanette Stroebele-Benschop

**Affiliations:** 1 Department of Applied Nutritional Psycholoy, Institute of Nutritional Medicine, University of Hohenheim, Fruwirthstr. 12, 70593 Stuttgart, Germany; 2 Daimler AG, Leibnitzstr. 2, 71032 Böblingen, Germany

**Keywords:** Truck drivers, Food choices, Eating behaviour, Diet, Occupational health, Overweight/obesity

## Abstract

**Background:**

Long-haul truck drivers are exposed to unfavorable working conditions affecting their health but information on truck drivers travelling through Europe is missing. The study aimed to describe the populations’ characteristics and food choice patterns while working compared with eating patterns at home, taking weight status into account.

**Methods:**

A cross-sectional survey using questionnaires in 12 languages conducted at two truck stops in Germany.

**Results:**

Among 404 truck drivers of 24 nationalities, only 24% were normal weight while 46% were considered overweight and 30% obese. In regards to their health, more than half reported that they smoked and 32% reported at least one chronic disease. 37% ate their meals often or always at truck stops, while 6% never did so. The most common food items brought from home were fruits (62%) followed by sausages (50.6%), sandwiches (38.7%), self-cooked meals (37%), sweets (35.4%), and raw vegetables (31%). Bivariate analyses revealed differences in food choices during work and at home with more sausages, energy drinks and soft drinks, and canned foods eaten during trips. Fresh vegetables, legumes and fish were more often chosen at home. Available food appliances in trucks appeared to be associated with food choice patterns. Interestingly, food choice patterns and food preparation did not differ significantly across weight categories.

**Conclusions:**

The working conditions of professional truck drivers make a healthy lifestyle difficult to follow and appear to influence food choices while working. Particular effort should be taken to improve food choice patterns, food preparation and purchasing possibilities during trips.

## Background

In 2013, almost 550.000 professional truck drivers were registered in Germany [1]. The percentage of trucks as freight traffic continuously rises and is currently at over 70% in the country [2]. Germany is an important Europe-wide transportation hub with 33.6 billion kilometers driven on the motorway and major toll roads by German and foreign trucks in 2017 [3]. However, professional truck drivers are a particularly at-risk population in many health aspects given their stressful and unfavourable working conditions [4].

While the need for truck drivers is increasing, the working conditions have only slightly improved over the years. Truck drivers often report sleep problems and back pain as well as other physical health problems [5–7]. Behavioural health problems include alcohol abuse, anxiety, depression, job strain, fatigue, and social isolation [6, 8]. In addition, high prevalence rates of overweight, obesity, inactivity, and unhealthy eating patterns are observed among this population [9, 10]. Recent studies with long-haul truck drivers in the U.S. revealed higher levels of cholesterol, blood pressure, obesity, and diabetes as well as higher smoking rates than the U.S. average [11, 12]. Obesity in truck drivers has been linked to truck accidents in the U.S. [13]. Long distance truck drivers in North West England reported low levels of daily fruit and vegetable intake as well as low levels of exercise accompanied with high levels of obesity and smoking [10]. Overall, fruit and vegetable intake seems low while fat and total caloric intake seems high in this particular population [14, 15].

Given their working conditions, with regulated breaks and driving hours, truck drivers often snack or eat their meals while driving or at truck stops, which limits their available food choices. The truck itself only has limited space for a refrigerator, storage and cooking space (or portable cooking tools) which further restricts the transportation and preparation of fresh and healthy products. Truck stops only offer inadequate amounts of healthy food options and the purchase of fresh fruits and vegetables is rarely possible. Parking regulations and vehicle size often prevent drivers from going grocery shopping while “on tour” and only a few truck drivers seem to try to incorporate physical activity while “on tour” [16]. Interestingly, very little is known about eating behaviour patterns in relation to the working conditions of European truck drivers driving through Europe [17].

Furthermore, existing studies examined dietary patterns of truck drivers but did not determine whether food choices differ between work and home [14, 15, 18]. More specific and tailored information regarding the types of food consumed while working and potential differences in food choice patterns at home and at work among long distance truck drivers has not been gathered. Thus, the aim of this study was to take a look at the socio-demographic characteristics of truck drivers driving through Germany and to examine food choice patterns and their correlates among this population taking BMI into account.

## Methods

The study was designed as a cross-sectional survey among long-haul truck drivers driving through Germany. Data were assessed based on a self-developed self-report questionnaire since existing questionnaires lacked questions regarding specific food groups [10, 14, 15, 18] or were too exhaustive for an on-site survey (e.g. 24-h recalls) [19].

### Measures

The questionnaire was developed in German based on expert interviews and existing pilot work [19, 20] and pretested in April 2018 at one truck stop. Based on the pretest experience, the questionnaire was improved (e.g. shortened, reworded and better categorized) and then translated into eleven additional languages by a professional translation service, according to common countries of origin of the population of interest. Besides socio-demographic information, food choice patterns at home and during work for snacking and main meals (e.g., where do you eat your meals while on tour? What do you eat while on tour and while at home/outside of work? How often do you eat a meal at the truck stops while on tour?), available food storage and preparation equipment in the truck, and health status questions including smoking, alcohol consumption and diagnosed chronic illnesses were included (Table 1).

**Table 1:** Summary of the self-report questions used in the survey

Variables	Answer categories/−scales
age, weight, height	insert number
gender	male/female
nationality	insert
“Do you smoke?“	yes/no
“How many consecutive nights on average do you not spend at home during a job?”	0, 1, 2, 3, 4, 5, 6, 7, 8, 9, 10, 11, 12, 13, 14, > 14
“Do you bring food with you on your trips?”	yes/no
“If you bring food with you, what kind do you normally bring?”	multiple answers possible: soft drinks/energy drinks, juices/spritzers, water, self-prepared warm meal, warm ready-made meals, sandwiches, sausage, noodle/potato/meat salad, green salad, fruit, raw vegetables, chips/crackers/pretzel sticks, nuts, sweets, other
“Please indicate whether you eat the following foods more often at home or during work”, e.g. fried products, fresh vegetables, canned food, …	more often at work, same, more often at home, not at all
“How often do you visit fast food chains and/or snack stands during work/during leisure time?	never, rarely, sometimes, often, always
“How often do you normally eat a main meal from a service station/truck stop?”	never, rarely, sometimes, often, always
“If you eat meals from service stations/truck stops, which dish do you prefer?”	meat/sausage dish, fish dish, vegetarian dish, salad, sandwich, dessert/cookies
“How much alcohol do you drink on average per week?”	amount of beer (0.5 l)/wine (0.2 l)/schnaps (2 cl)
“Do you have a chronic illness diagnosed by a doctor?”	multiple answers possible: I do not have a chronic illness, Diabetes type 1, Diabetes type 2, high blood pressure, gastrointestinal illnesses, rheumatism, asthma, gout, chronic back pain, other (indicate)
“If you bring food with you, where do you eat it?”	multiple answers possible: In the truck during the trip; In the truck during breaks; At the service station/service area/truck stop (seating opportunity)
“Do you have the following appliances to store, prepare, or reheat food in the truck?”	multiple answers possible: refrigerator, kettle, coffee machine, gas cooker, microwave, I have none of these appliances

### Procedures

Participants were recruited on two autobahn service areas in southern Germany on 17 days in May and June 2018 by two trained researchers. Truck drivers were approached near their trucks and asked to fill out the questionnaire. If they did not understand German, they could choose a questionnaire in the appropriate language if available by pointing at the country flag. The questionnaire was handed over in the respective language, and filled out by the participants. As a thank you gift, they could choose between a baseball cap or a travel mug upon questionnaire completion. Informed consent was obtained by a checkbox on the front of the questionnaire below general information about the project. The study was approved by the ethics committee of the University of Hohenheim.

### Data analysis

Descriptive statistics are used to report the prevalence of various factors. Categorical data are presented as numbers and percent, continuous data as mean ± standard deviation (SD).

Bivariate associations between categorical variables were tested by chi square, or Fisher’s exact test where required (weight status groups with availability of gas cooker/microwave, cooler box/fridge, and weight status groups with chronic diseases). Mean differences between two groups were tested by independent t-tests (food product consumption at home vs. at work, frequency of truck stop and fast food chain/snack stand visits). Associations between continuous variables are presented as Pearson correlations (BMI and frequency of fast food visits). Due to the low total number of female respondents (*n* = 7), data were not analysed for gender differences. The number of respondents can differ from the total sample of 404 valid cases due to missing replies in single items. Data were entered and analysed using IBM SPSS Statistics, Version 25. BMI is reported in kg/m^2^ and classified using cut-offs by the World Health Organization [21].

## Results

In total, 419 truck drivers completed the questionnaire, which resulted in a response rate of 69.5%. Fifteen truck drivers were excluded because they only worked short range distances without spending nights away from home; 404 truck drivers were included in the data analysis. Overall, about half of the participants (53%) stated they generally spent more than five days on tour. Almost one third (33%) spent more than 14 nights away from home on an average tour.

Personal characteristics of the 404 truck drivers can be found in Table 2. At 33%, the majority of the drivers were German, however, almost 50% of the drivers were from Eastern-European countries. More than half of the sample indicated that they smoked. Almost 46% were considered overweight and 30% were considered obese.

**Table 2:** Sample characteristics (percentage or mean ± SD of valid indications; *n* = 404)

	*n (%)*	*mean ± SD*
*gender*		
male	394 (98)	
female	7 (2)	
age		45.2 ± 10.9
*questionnaire language*		
German	165 (41)	
Polish	47 (12)	
Romanian	47 (12)	
Russian	36 (9)	
Bulgarian	28 (7)	
Hungarian	25 (6)	
Dutch	16 (4)	
English	13 (3)	
Serbian	11 (3)	
Turkish	7 (2)	
Slovenian	6 (1)	
Slovakian	3 (1)	
*nationality/citizenship*		
German	127 (33)	
Romanian	43 (11)	
Polish	41 (11)	
Dutch	29 (8)	
Bulgarian	25 (7)	
Hungarian	25 (7)	
Serbian	15 (4)	
Ukrainian	13 (3)	
other	62 (16)	
Body Mass Index (BMI; kg/m^2^)		28.4 ± 4.8
*BMI (body mass index) classes*		
normal weight (18.5 ≤ BMI < 25)	90 (24)	
overweight (25 ≤ BMI < 30)	169 (46)	
obese (BMI ≥ 30)	111 (30)	
*smoking*		
yes	215 (54)	
no	185 (46)	
*school education*		
no graduation	8 (2)	
graduation at 9th grade	111 (28)	
graduation at 10th grade	114 (29)	
graduation at 12th/13th grade (university entrance qualification)	134 (34)	
other	25 (6)	
*trucking company location*		
Germany	157 (39)	
European Union (except Germany)	224 (56)	
non-EU country	17 (4)	

Smokers were more often normal weight (30%) compared to non-smokers (18%). 77% of the normal weight truck drivers reported no chronic disease compared to 59% of the obese truck drivers and 71% of the overweight truck drivers. Apparent differences were found in those diagnosed with a chronic disease. Of the obese truck drivers, 8% reported being diagnosed with diabetes type 2 while only 2% of the overweight drivers indicated having diabetes type 2 and none of the normal weight drivers (χ^2^(2) = 12.123, *p* = 0.002). High blood pressure was a health concern for 23% of the obese truck drivers, but only by 9% of the overweight and 7% of the normal weight drivers (χ^2^(2) = 14.310, *p* = 0.001). Chronic back pain, however, was more evenly distributed with 7% of normal weight, 9% of overweight, and 10% of obese truck drivers reporting chronic pain in their back (χ^2^(2) = 0.657, *p* = 0.720).

Drivers indicated to drink on average 5.1 ± 6.6 units of alcohol per week. Beer was drunk most often (3.7 ± 4.9 units of 500 ml), compared to schnaps (1.1 ± 4.2 units of 20 ml) and wine (0.7 ± 1.9 units of 200 ml). One quarter of the drivers stated drinking less than one serving of alcohol per week. There was no association with BMI.

### Food choice patterns at home and during work

The majority of the drivers reported eating their snacks or meals from home in their truck (73%) and a third reported doing so at the truck stop or restaurant (33%). Self-prepared meals were taken on tour by 36% of the sample while 29% indicated taking ready-to-eat meals (convenience food) and 38% indicated taking sandwiches along. Almost half of the participants brought sausages on trips and obese truck drivers (56%) did so more often when compared to normal weight (46%) or overweight drivers (49%), albeit not significantly (*p* = 0.318). Fresh raw vegetables were brought from home by 30% and fresh fruit by about 60% of the participants. Bringing salty snacks was only reported by 13% and sweets by 34%.

A third of the participants reported sometimes eating their main meal at the truck stop restaurant (32%) and 37% reported to do so often or always. If the meal was eaten at the restaurant, the majority of the drivers chose a meal with meat (77%) or fish (12%). The remaining 12% chose vegetarian meals (2%), salads (6%), sandwiches (3%) or desserts (1%).

**Fig. 1:**
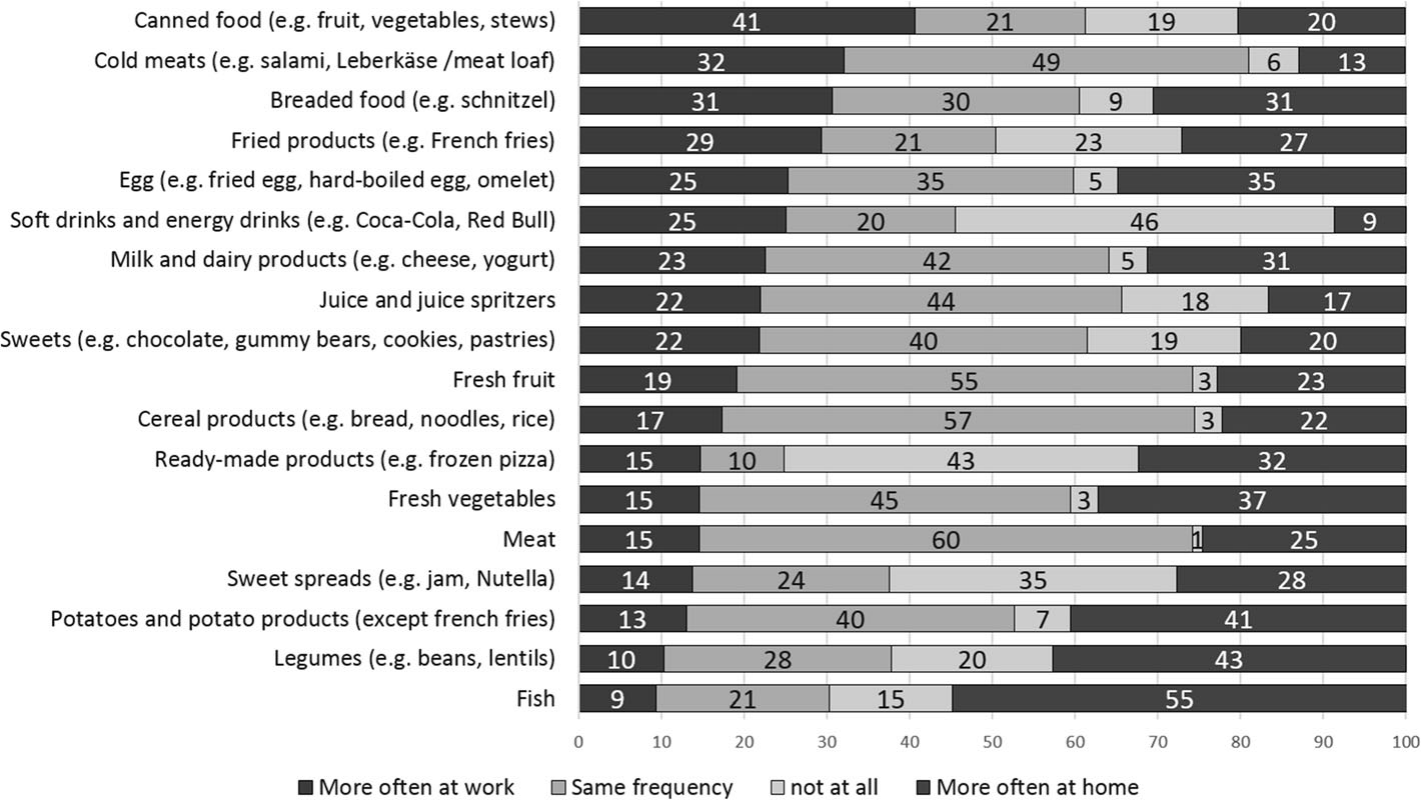
Frequency of consumption of selected food categories (in %) comparing consumption location (at home vs. at work)

Differences between food choices while on tour compared to when at home or off work were found (Fig. 1). Energy drinks (25% vs. 8.7%), sausage products (32.1% vs. 12.8), and canned foods (40.7% vs. 20.2%) were reported to be eaten or drunk more often on tour compared to when at home, while this seemed to be reversed for meat (14.6% vs. 24.6%), milk and dairy products (22.6. vs. 31.3%), fresh vegetables (14.6% vs. 37.3%), legumes (10.3% vs. 42.7%) and fish (9.4% vs. 54.9%). Truck drivers visited fast food chains and snack stands more often during leisure time (t(356) = − 2.787, *p* = 0.006) than while on tour. The association between fast food chain visits and BMI is stronger for fast food visits during leisure time (r = 0.107, *p* = 0.050), than for fast food visits during work (r = 0.086, *p* = 0.107). No significant associations between BMI or weight categories and other food choice patterns were found.

### Availability of food storage and preparation equipment

Cooler boxes or refrigerators were available in the trucks of 94% of the participants. About a third indicated that they had a water boiler (33%) or coffee machine (35%) in their truck. A gas cooker on board was reported by 62% and only 8% reported a microwave in their vehicle. The availability of appliances to store and prepare food also seemed to influence eating behaviour in terms of the types of food taken on trips (Table 3) but was not significantly associated with BMI. Drivers who had a gas cooker or microwave ate less often at truck stops (2.98 vs. 3.38 on a 5-point Likert-scale from 1 = never to 5 = always; t(392) = − 3.674, *p* < 0.001).

**Table 3:** Number of “yes“ answers [n (%)] for the brought food items (“If you bring food with you, which food items do you bring?”) separated by food storage and preparation equipment

	Do you have the following appliances in your truck?					
	gas cooker or microwave			cooler box or fridge		
Food items brought on the trip	available (*n* = 265)	not available (*n* = 127)	p	available (*n* = 367)	not available (*n* = 25)	p
soft drinks, energy drinks	60 (23)	23 (18)	0.30	79 (22)	4 (16)	0.51
juices, spritzers	89 (34)	45 (35)	0.72	127 (35)	7 (28)	0.50
water	209 (79)	105 (83)	0.38	297 (81)	17 (68)	0.12^f^
personally prepared warm meal beforehand (e.g. soups, stew, pasta)	122 (46)	23 (18)	< 0.01	140 (38)	5 (20)	0.07
warm ready-made meal (e.g. soups, stew, pasta)	93 (35)	23 (18)	< 0.01	112 (31)	4 (16)	0.12
sandwiches	87 (33)	65 (52)	< 0.01	144 (39)	8 (32)	0.47
sausages (e.g. smoked, Wiener)	146 (55)	52 (41)	< 0.01	193 (53)	5 (20)	< 0.01
pasta, potato, meat salads	62 (23)	24 (19)	0.31	81 (22)	5 (20)	0.81
green salads	64 (24)	15 (12)	< 0.01	78 (21)	1 (4)	0.04
fruit	168 (63)	75 (59)	0.41	236 (64)	7 (28)	< 0.01
raw vegetables	91 (34)	31 (24)	< 0.05	117 (32)	5 (20)	0.21
salty snacks (e.g. chips, crackers, pretzels)	40 (15)	13 (10)	0.19	51 (14)	2 (8)	0.55^f^
nuts	38 (14)	13 (10)	0.26	49 (13)	2 (8)	0.76^f^
sweets	95 (36)	44 (35)	0.82	134 (37)	5 (20)	0.09

*p*-values based on χ^2^ or ^f^Fisher’s exact test

## Discussion

Truck drivers face difficult working conditions including many health and social challenges. Their limited access to food, specifically balanced food choices, and their isolated and small work place are just two examples of the challenging work setting. This survey confirmed the results of other studies regarding an above average BMI in the truck driver population [7, 22] and a high prevalence of bad health conditions such as back pain or other chronic diseases; particularly in obese truck drivers [9, 23]. European truck drivers driving through Germany appear to be affected by their difficult working conditions in similar ways than drivers in other European countries, the U.S., or Australia [9, 17, 23–25].

Interestingly, overweight and obese truck drivers did not seem to differ significantly in their food choice patterns which was also found by Whitfield Jacobson and colleagues [14] among U.S. truck drivers. However, neither their study nor this study assessed the amount of food consumed.

To our knowledge, this is the first study that focused on food choice patterns of truck drivers during trips compared to at home food choices. Several novel findings should be highlighted. The food choices at work seem to differ unfavorably from the choices when at home, such as intake of sausages or energy drinks and soft drinks. While sausages are convenient to consume while traveling, processed meat found in sausages is usually high in fat and high intake of processed meat, such as sausages, has been linked to increased risk of coronary heart disease as well as different types of cancer [26–28]. The higher intake of soft drinks and energy drinks while on tour is not surprising given the tiresome and exhausting nature of driving for many hours with only short breaks. However, while there seems to be no differences in the BMI groups in the frequency of drinking soft drinks or energy drinks, the consumption of energy and soft drinks has been associated with negative health consequences such as type 2 diabetes and cardiovascular complications [29], or in the case of energy drinks, sleep disturbances [30, 31]. Fresh vegetables on the other hand were more often chosen at home. The lack of fresh vegetables while on tour has been mentioned in other studies as a barrier to healthy eating [16].

The study adds several new aspects to the already existing literature that need to be further investigated. It is the first study to separate eating occasions between work and home revealing differences in food choice patterns across those two occasions, which could help when developing interventions and campaigns to improve dietary habits with a focus on the foods taken on trips. With this knowledge, developing and implementing new strategies to promote healthy and balanced eating among this population can be undertaken. For instance, European supermarkets more than ever offer a wide variety of fresh ready-to-eat vegetable snacks that could easily be stored in the truck fridges for a couple of days. However, while this option could reduce one of the barriers to eating healthy foods on the road, purchasing or preparing food before work trips might be difficult for the drivers to organize, given their work schedule (e.g. leaving very early in the morning; leaving on Sunday when shops are closed in most of Europe).

In addition, having food related appliances on board appears to support healthier food options such as self-prepared meals, salads, or raw vegetables. Appliances, such as a gas cooker or microwave, provide the option to prepare healthy foods on tour that are difficult or even impossible to obtain at truck stops or restaurants.

Besides innovative behavioural interventions to improve health and body weight among this population, and healthier food items at truck stops, it is also important to look into possible advances in cabin environments by automobile companies, such as improved appliances that facilitate food self-preparation, storage, and cooking possibilities.

### Study limitations

This study has some limitations, such as its inability to determine causal relationships between studied variables and the questionnaire design, which might have distorted results. For example, self-reported body weight or alcohol consumption are both subject to social desirability. Furthermore, the fact that food quantities were not assessed reduces the study’s informative value. One positive aspect is that commercial truck drivers are notoriously difficult to reach and our study procedures allowed us to successfully enroll a large sample of truck drivers that travel regularly for days at a time across Europe.

## Conclusions

In conclusion, the study reveals differences in food choice patterns of truck drivers while on tour compared to while at home, which opens up opportunities for improving their dietary habits while at work using, for example, existing food storage and preparation appliances. In addition, educating truck drivers and presenting ways to improve their diet during trips by offering small changes, such as the purchase of snack vegetables or healthy precooked products available in stores to only be heated up on the gas cooker, might support dietary changes in this particular population.

## Data Availability

The dataset generated and analysed during the current study and the used questionnaire are available from the corresponding author on reasonable request.

## Abbreviations

BMI: Body mass index
SD: Standard deviation
